# Role of choledochotomy after mechanical lithotripsy‐related adverse event in endoscopic retrograde cholangiopancreatography

**DOI:** 10.1002/ccr3.7248

**Published:** 2023-04-23

**Authors:** Raquel Lalanda, Maria Inês Seixo, Ana Sofia Lopes, David Aparício, José Ferreira, Carlos Freitas, Luís Miranda

**Affiliations:** ^1^ General Surgery Department Centro Hospitalar Universitário Lisboa Norte EPE, Hospital de Santa Maria Lisbon Portugal; ^2^ Gastroenterology Department Centro Hospitalar Universitário Lisboa Norte EPE, Hospital de Santa Maria Lisbon Portugal

**Keywords:** choledochotomy, Dormia basket, endoscopic retrograde cholangiopancreatography (ERCP), mechanical lithotripsy‐related adverse event

## Abstract

In case of rupture of the mechanical lithotripter's traction wires during an ERCP, we suggest performing a choledochotomy to remove the stone, and remove the closed Dormia basket through the mouth.

## Short Abstract

Patient with choledocholithiasis underwent endoscopic retrograde cholangiopancreatography and the Dormia basket advanced within the common bile duct became impacted with an entrapped stone. A mechanical lithotripter was used to fracture the stone, but the wires of the basket ruptured. Choledochotomy was performed to successfully treat this mechanical lithotripsy‐related complication.

## 1 CASE PRESENTATION

An 83‐year‐old woman with hypertension and dyslipidaemia went to the emergency department with right upper quadrant pain associated with nausea and vomiting. On physical examination, she was afebrile, with normal vital signs and no abdominal relevant findings on clinical examination. Initial examination in the emergency department revealed a white blood cell count of 5.4 × 10^9^/L with CRP of 31.9 mg/dL, total serum bilirubin of 2.4 mg/dL, direct bilirubin 2.1 mg/dL, ALP 393 IU/L, GGT 879 IU/L, AST 61 IU/L, ALT 94 IU/L, and normal serum creatinine. Abdominal ultrasonography showed a mildly thickened gallbladder with multiple echogenic structures with acoustic shadows suggestive of calculi, and dilated intrahepatic and extrahepatic bile ducts (the common bile duct was 24 mm in diameter) that also contained multiple calculi. She was diagnosed with choledocholithiasis without cholangitis and underwent endoscopic retrograde cholangiopancreatography (ERCP).

**FIGURE 1:**
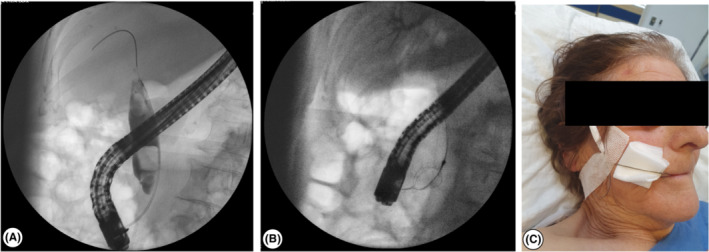
(A) Cholangiography with choledocholithiasis; (B) Basket with the impacted stone in the common bile duct; (C) The cut edge of the basket handle can be seen emerging from the mouth of the patient, following failed removal of the basket.

Endoscopic retrograde cholangiopancreatography revealed a dilated common bile duct with choledocholithiasis. A wide sphincterotomy was performed and while trying to remove the stones, with a Dormia basket, it became impacted with an entrapped stone of more than 10 mm in diameter. A mechanical lithotripter (Soehendra) was used to fracture the stone, but the traction wires broke near the patient's mouth (Figure 1).

## 2 QUIZ QUESTION: HOW TO PROCEED AFTER A LITHOTRIPSY‐RELATED ADVERSE EVENT WITHOUT AN ENDOSCOPIC SOLUTION?

**FIGURE 2:**
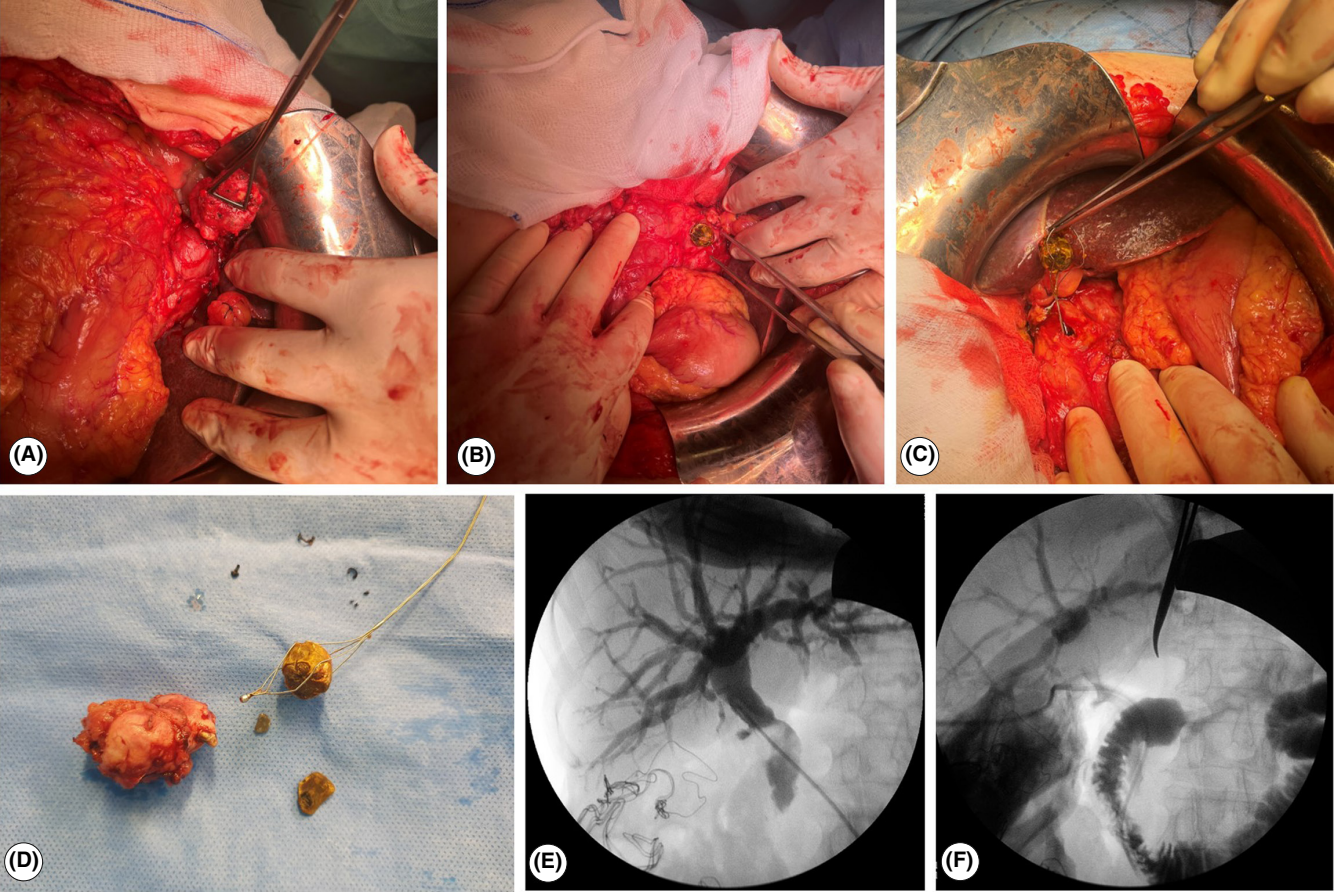
(A) Cholecystectomy; (B) Choledochotomy; (C) Extraction of the metal wires and the basket after performing choledochotomy; (D) Cholecystectomy specimen, successfully removed stone and Dormia basket; (E) Intraoperative cholangiography with opacified biliary tree; (F) Intraoperative cholangiography with opacified duodenum.

An exploratory laparotomy, adhesiolysis, Kocher maneuver, and cholecystectomy were performed. We performed a choledochotomy and pulled the impacted basket wires together with the stone out of the choledochal duct (Figure 2). We removed the impacted stone and tied up the basket wires with a 2/0 silk suture and pulled the basket out through the mouth, in order to prevent additional lesions on the gastrointestinal tract. High‐pressure lavage of the common bile duct allowed additional small biliary stones to be extracted. Intraoperative cholangiography demonstrated there were no more stones inside the biliary tract (Figure 2). The choledochotomy was closed by a 5/0 polydioxanone simple interrupted suture. After surgery, markers of cholestasis decreased, and patient was discharged at 5th postoperative day.

Gallstones affect 10%–15% of adult population in developed countries.^1^ Patients develop symptoms and/or complications in 25% of cases, and these might be severe in 1%–2%.^1^ Up to 10%–15% of biliary stone extraction procedures are demanding and require additional endoscopic techniques to allow stone clearance.^2^ Technical difficulty of common bile duct endoscopic clearance is influenced by different factors: patient's clinical condition, the stone's characteristics, and anatomical factors.^1^

Mechanical lithotripsy is an effective procedure, with a success rate ranging between 76% and 91%.^1^ Retrospective studies report some lithotripsy‐related adverse effects: trapped/broken baskets, traction wire fracture, broken handle and perforation/duct injury.^1^ The great majority of these complications are treated endoscopically with salvage or alternative lithotripsy techniques, extension of sphincterotomy or stent placement, but occasionally surgery is the only available option.^1^

Some studies report open bile duct surgery to be superior to ERCP in achieving common bile duct stone clearance.^3^ However, management always needs to be tailored to the patient's characteristics and anatomical conditions.^2^ In case of a lithotripsy‐related adverse event without an endoscopic solution, we suggest performing exploratory laparotomy, followed by choledochotomy to remove the stone, and remove the closed Dormia basket through the mouth.

## AUTHOR CONTRIBUTIONS

**Raquel Lalanda:** Conceptualization; formal analysis; investigation; methodology; resources; software; validation; visualization; writing – original draft; writing – review and editing. **Maria Inês Seixo:** Validation; visualization; writing – review and editing. **Ana Sofia Lopes:** Validation; visualization; writing – review and editing. **David Aparicio:** Conceptualization; formal analysis; supervision; validation; visualization; writing – review and editing. **José Ferreira:** Supervision; validation; visualization. **Carlos Freitas:** Supervision; validation; visualization. **Luís Miranda:** Supervision; validation; visualization.

## CONFLICT OF INTEREST STATEMENT

All authors declare no conflict of interests.

## CONSENT

Written informed consent was obtained from the patient to publish this case report in accordance with the journal's patient consent policy.

## Data Availability

The data that support the findings of this study are available from the corresponding author upon reasonable request.
